# Short-term increase in discs’ apparent diffusion is associated with pain and mobility improvements after spinal mobilization for low back pain

**DOI:** 10.1038/s41598-018-26697-7

**Published:** 2018-05-29

**Authors:** Paul Thiry, François Reumont, Jean-Michel Brismée, Frédéric Dierick

**Affiliations:** 1 OMT Skills, Private physical therapy practice, La Louvière, 7100 Belgium; 2 Center for Rehabilitation Research and Department of Rehabilitation Sciences, Texas Tech University Health Sciences Center, Lubbock, Texas USA; 3 Forme & Fonctionnement Humain Lab, Physical Therapy Department, CERISIC, Haute Ecole Louvain en Hainaut, Montignies-sur-Sambre, 6061 Belgium; 4 Université catholique de Louvain, Faculty of Motor Sciences, Louvain-la-Neuve, 1348 Belgium

## Abstract

Pain perception, trunk mobility and apparent diffusion coefficient (*ADC*) within all lumbar intervertebral discs (IVDs) were collected before and shortly after posterior-to-anterior (PA) mobilizations in 16 adults with acute low back pain. Using a pragmatic approach, a trained orthopaedic manual physical therapist applied PA mobilizations to the participants’ spine, in accordance with his examination findings. *ADC*_*all*_ was computed from diffusion maps as the mean of anterior (*ADC*_*ant*_), middle (*ADC*_*mid*_), and posterior (*ADC*_*post*_) portions of the IVD. After mobilization, pain ratings and trunk mobility were significantly improved and a significant increase in *ADC*_*all*_ values was observed. The greatest *ADC*_*all*_ changes were observed at the L_3_-L_4_ and L_4_-L_5_ levels and were mainly explained by changes in *ADC*_*ant*_ and *ADC*_*post*_, respectively. No significant changes in *ADC* were observed at L_5_-S_1_ level. The reduction in pain and largest changes in *ADC* observed at the periphery of the hyperintense IVD region suggest that increased peripheral random motion of water molecules is implicated in the IVD nociceptive response modulation. Additionally, *ADC* changes were observed at remote IVD anatomical levels that did not coincide with the PA spinal mobilization application level.

## Introduction

Among all musculoskeletal pain conditions, the prevalence and burden from low back pain (LBP) [ICD-10-CM, code M54.5] is high throughout the world. Of the 291 conditions studied in the Global Burden of Disease 2010 study, LBP ranked highest in terms of disability and sixth in terms of overall burden^1^. Posterior-to-anterior (PA) spinal mobilization is a common^2^ and relatively safe physical therapy intervention^3^ to treat LBP. It can result in immediate detectable improvements in pain^2^ and restoration of movement functions^4^. However, despite the widespread use of lumbar spinal mobilization, the physiological responses of lumbar anatomical structures are still largely unknown. Recent advances in musculoskeletal magnetic resonance imaging (MRI) allow free water movement observation within and between tissues *in vivo*, and is called diffusion-weighted (DW) MRI. This emerging imaging technology is particularly sensitive to small changes in fluid flow and has a great potential for studying the influence of physical therapy interventions such as manual therapy, exercise, and physical agents on musculoskeletal structures^5^.

Based on the comparison between DW images and non-DW images using the same MRI sequence, it is possible to reconstruct diffusion mapping and calculate an apparent diffusion coefficient ($ADC$) within the intervertebral disc (IVD)^6–9^. The $ADC$ measures *in vivo* the amount of water protons diffusion (Brownian microscopic motion) within a voxel of tissue, and are expressed in units of $m{m}^{2}\,{s}^{-1}$ ^6,8,10^. Interestingly, IVD DW MRI has been successfully used for some years by Beattie and his colleagues^10–13^. It allowed to link the decreasing pain reported by participants with chronic LBP following single session of lumbar PA mobilizations from L_5_ to L_1_ levels associated to McKenzie prone press-ups^14^, to the increase in lumbar IVD $ADC$ values^12^ or high-velocity, short-amplitude thrusts at L_5_-S_1_ level^13^. From a physiological point of view, water diffusion within the IVD has been suggested as one mechanism of analgesia following manual mobilization/manipulation^5^, but the complete mechanism is still unknown.

Despite the exciting and innovative natures of the studies that explored simultaneously $ADC$ in IVD and pain changes after spinal mobilization/manipulation in LBP patients, different methodological choices may have influenced the results and made it difficult to generalize to clinical settings. *First*, selected investigations included young patients only^12,13^ and consisted of prescribed mobilization/manipulation in LBP patients with heterogeneous symptoms chronicity and intensity^12^. However, with advancing age, vascular disease becomes more prevalent and a significant association between atherosclerotic lesions in abdominal aorta and LBP exists^15^. The abdominal aorta in people with LBP is affected by atherosclerosis more frequently than in people without LBP. Such phenomenon could explain degenerative IVD disease because of the resulting decrease IVD nutrition by insufficient blood supply from the lumbar arteries^15,16^. *Second*, $ADC$ values were only computed in the IVD central portion. It is therefore necessary to compute values in adjacent IVD central portion regions to better understand its global physiological response. Indeed, the IVD is a heterogeneous structure, at both the macroscopic and microscopic levels, which could induce inhomogeneous variations in water diffusion content according to anatomical regions^17^ other than the *nucleus pulposus* (NP) explored in previous studies^12,13^.

Today, a more pragmatic trial investigating the effect of spinal mobilizations on lumbar IVD $ADC$, pain perception and trunk mobility changes is needed. It is clinically relevant to associate changes in trunk mobility with changes in pain and IVD water diffusion since trunk altered general or segmental kinematic behavior, whether restricted, excessive, or linked to poor motor control, is associated with LBP^18,19^ and their identification frequently guides the conservative therapeutic approach^19,20^. Degenerative IVD changes are associated with a dramatic loss of water content and height^21^. Dehydration can occur in the IVD from either the loss of NP pressurization or tissue overloading^21^ and strongly influences IVD tissue biomechanics^22,23^ that may, in turn, alter segmental kinematic. Therefore, we conducted a single group before and after intervention with the objective to better understand the short-term effect of PA mobilizations applied to vertebrae on lumbar IVD $ADC$, pain perception and trunk mobility changes in participants suffering from idiopathic acute LBP. Contrary to previous studies using DW MRI to assess the IVD physiological response from a single region of interest (ROI), $ADC$ maps were computed in 9 ROIs in the IVD center and relationships between $ADC$, pain perception and trunk mobility changes were explored.

## Methods

### Subjects

A convenience sample of 16 adult patients (11 women and 5 men) was consecutively recruited during a 6 months period (January 2015 to June 2015) from a private physical therapy practice (OMT Skills, La Louvière, Belgium), with complaints of acute idiopathic LBP diagnosed by a physician; age: 46 ± 16 years (range: 26–85), height: 165.8 ± 9 $cm$, weight: 73.4 ± 17 $kg$, and body mass index: 26.6 ± 4 $kg\,{m}^{-2}$. Participants’ inclusion and exclusion criteria were similar to previous studies^24,25^. Inclusion criteria were: aged between 20 to 85 years, suffering from acute LBP (<6 weeks of pain) with stiffness, asymptomatic for at least one month between the current and previous LBP episodes, and reports of more days without pain than days with pain in the past year. Exclusion criteria were: aversion to spinal mobilization, chronic LBP, radiating pain below the knees, spine fracture or surgery, osteoporosis, pregnancy, implanted devices that could interact with the MRI magnetic field, claustrophobia, obesity, alcohol or drug abuse, mental illness or lack of cognitive ability.

A priori sample size estimation was carried out by using G*Power software (Version 3.1.9.2), with an *α* level (I) equal to 0.05 and *β* level (II) equal to 0.20, with a statistical power of 0.80. The estimation was made on the basis of the average results obtained by Beattie *et al*.^13^ who reported a significant $ADC$ increase at the L_1_-L_2_ IVD (1.70 ± 0.25 × 10^−3^ $m{m}^{2}\,{s}^{-1}$ *versus* 1.80 ± 0.24 × 10^−3^ $m{m}^{2}\,{s}^{-1}$) after lumbar PA mobilization in young participants with low intensity LBP. A 0.41 effect size $dz$ was calculated for unilateral t test for paired samples and a 0.5 correlation between the groups. The total sample size estimate was 39. However, we stopped the participants’ recruitment before reaching the target sample size.

The study protocol and the informed consent documents were approved by the medical ethics committee of the Université catholique de Louvain (2014/07AOU/419) – Belgian registration nr = B403201421675; reference number on BioMed Central: ISRCTN16069685 DOI 10.1186/ISRCTN16069685. All research was performed in accordance with relevant guidelines/regulations, and informed consent was obtained from all participants.

### General procedure

Before study participation, all procedures were explained to the participants. One investigator (R.F.) invited the participants to complete a visual analogue scale (VAS) for pain^26^, a DN4 (Douleur Neuropathique 4) questionnaire^27^, and a shortened version of McGill Pain Questionnaire validated in French (Questionnaire Douleur Saint-Antoine, QDSA)^28^. All outcome measures are valid^29,30^ and reliable^27,31^. The DN4 is a clinician-administered questionnaire consisting of 10 items for neuropathic pain screening. It has components regarding patient’s pain interpretation and includes hypoaesthesia and allodynia assessment. QDSA has 58 word descriptors categorized into 16 subgroups, including 9 sensory groups and 7 affective groups. The participants select the word descriptors and score them from 0 (not at all) to 4 (extremely). A sensory (QDSA-S), affective (QDSA-A), and total QDSA score (QDSA-T) were computed as the sum of A to I (/36), J to P (/28), and A to P items (/64), respectively. A second investigator (T.P.), blinded to the first investigator’s evaluations, invited the participants to evaluate their pain using an oral analogue scale (OAS) and performed various trunk mobility tests in standing posture: flexion [$TF$], extension [$TE$] and left and right lateral flexion [$TL{F}_{l}$ and $TL{F}_{r}$]. A neuro-dynamic test, the slump test^32^, was also conducted.

A first MRI scan of the participants’ lumbar region was then carried out. After this, a spinal Maitland’s PA mobilization^4^ was performed by another investigator (T.P.). The mobilization was performed in a consultation room, very close to the scanner (distance: 30 meters), and equipped with a classic medical examination table. A mechanical floor weighing scale (Seca 762, Hamburg, Germany) was placed under the feet of the orthopaedic manual physical therapist (OMPT) to record the change in weight exerted during PA mobilization. At this point in time, neither of the two investigators were informed of the results of the initial imaging. To complete data collection, a second MRI scan, identical to the first, was carried out within an hour after the spinal mobilization (5–50 $min$, 15 $\pm$ 10 $min$). Pain ratings and trunk mobility tests were again recorded by the two investigators. Total time of the procedure was around 90 $min$, including 2 × 12 $min$ for MRI, and 45 $min$ for physical examination (pain ratings and trunk mobility tests) and questionnaires (participants sat for approximately 30 $min$, half of the time before the first scan and half of the time before the second scan). The time spent in sitting position between the first and second MRI assessments was similar and the 30 meters that separated the treatment and MRI rooms were walked immediately after the sitting position for both MRI assessments. The duration of PA mobilizations was approximately 10 $min$ (Table 1).

**Table 1:** Characteristics of PA mobilizations applied for each participant, slump test results and pain medication.

Participants	PA level	PA location	PA grade	PA frequency	PA time	Slump	Pain medication
	1^st^ (2^nd^)			Hz	s	+/− (side)	
1	L_4_	↓	III	0.5–1	720	+ (left)	none
2	L_4_		III	0.5–1	720	−	paracetamol
3	L_5_		IV	1.5–2	450	+ (right)	NSAID
4	L_4_		IV	1.5–2	720	+ (right)	none
5	L_4_		IV	1.5–2	720	−	none
6	L_5_		IV	1.5–2	600	+ (left)	NSAID
7	L_3_	↓	IV	1.5–2	620	−	none
8	L_3_		III	0.5–1	720	−	paracetamol, NSAID
9	L_5_		III	0.5–1	630	+ (left)	paracetamol
10	L_5_		IV	1.5–2	417	+ (right)	NSAID
11	L_5_ (L_1_)	↓	IV	1.5–2	720	+ (left)	tramadol, paracetamol, NSAID
12	L_4_		IV	1.5–2	480	−	NSAID
13	L_3_ (T_11_)		IV	1.5–2	720	+ (right)	none
14	L_1_ (L_5_)		IV	1.5–2	720	+ (right)	none
15	L_4_		III	0.5–1	720	+ (right)	paracetamol
16	L_4_		III	0.5–1	720	−	none

1^st^ and 2^nd^ denote primary and secondary levels of PA mobilizations. PA grade III corresponds to a large amplitude movement that reaches the end range of movement (ROM), and grade IV to a small amplitude movement at the very end ROM, as defined by Maitland^4^. PA mobilizations frequency selected by OMPT was between 0.5–1 (1.5–2) Hz for grade III (IV). Slump test did not induce pain below the knees and was considered as positive (+) when pain increase was felt in the back, buttocks or thighs or negative (−) when pain did not increase. NSAID denotes non-steroidal anti-inflammatory drug.

↓Central application of mobilizations on the spinous process.

Unilateral application of mobilizations on left lamina.

Unilateral application of mobilizations on the right lamina.

### Physical examination and PA mobilization

The principal investigator (T.P.), a certified OMPT, with more than 30 years of experience performed the physical examination. It consisted of a complete orthopaedic manual therapy physical examination, inspired by Maitland’s physical examination^4^, and aimed to collect information, first subjective (interrogation) and then objective (physical assets), to confirm the origin of the participant’s lumbar pain symptoms. It also allowed the OMPT to reassess the participant following spinal mobilization. During trunk mobility tests ($TF$, $TE$, $TL{F}_{l}$, and $TL{F}_{r}$), a centimetric measure of major fingertip-to-floor distance was made before and after mobilization. For all measurements, the starting position was upright with the therapist’s foot width placed between the participants’ feet. Each participant was invited to lean the trunk forward ($TF$), backward ($TE$) or laterally ($TL{F}_{l}$ and $TL{F}_{r}$), without moving their feet, with their knees extended and their arms hanging freely under the action of gravity. Excellent intra-observer reliability of fingertip-to-floor measurements was reported for $TF$ (ICC = 0.98 to 0.99)^33,34^, $TLF$ (ICC = 0.98)^33^, but is unknown for $TE$.

**Figure 1:**
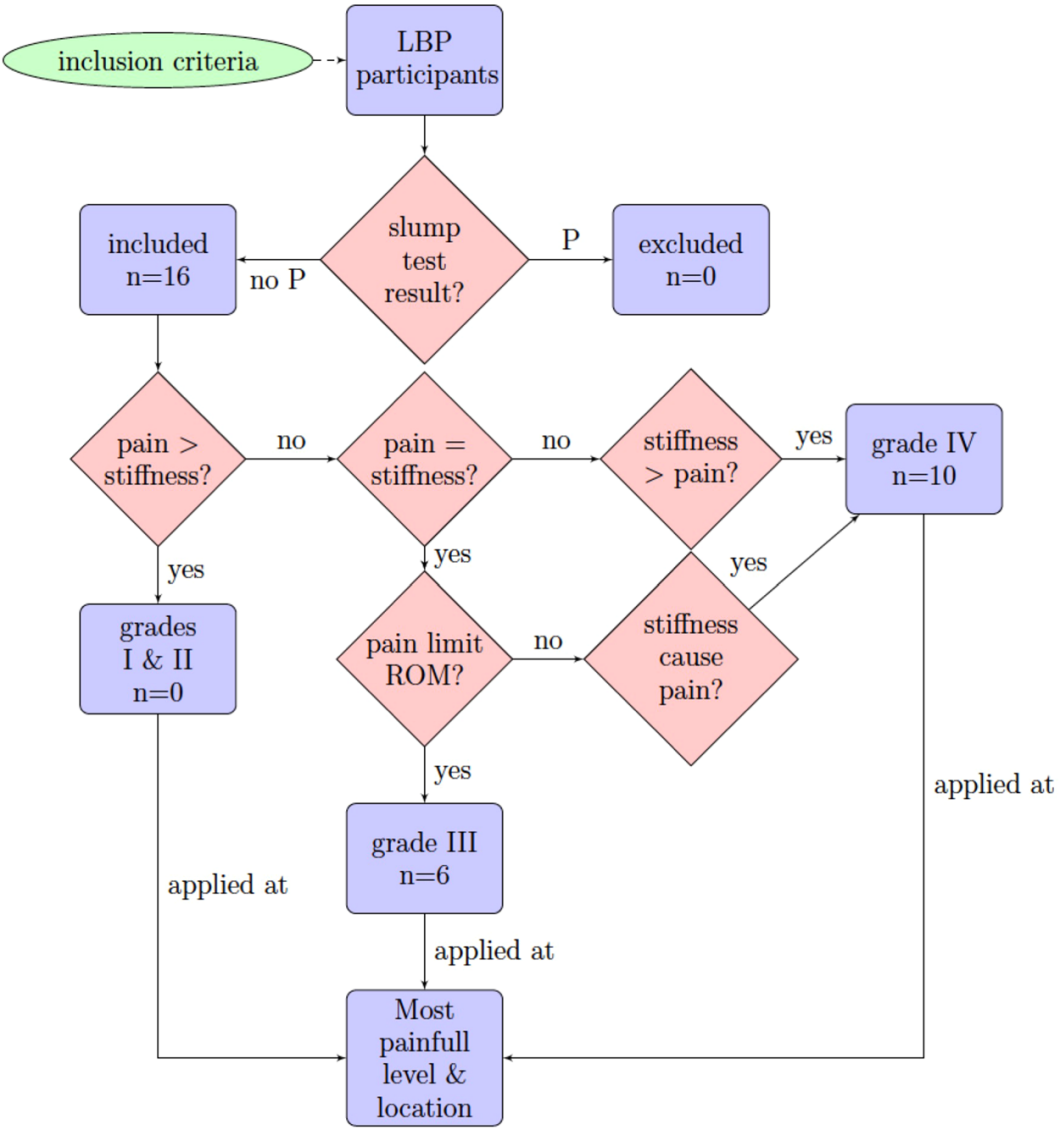
Decision tree used by the orthopaedic manual physical therapist (OMPT) to select the parameters of PA. Grades I & II corresponds to clinical group1, and grades III & IV to clinical 2 and 3, described by Maitland^4^. Here, only grades III and IV were selected by the OMPT, ranging from III− − to III+ + and IV− − to IV+ +. When pain was greater than stiffness, the OMPT felt pain as the limiting factor of movement early in the ROM; when pain was equal to stiffness, the OMPT felt pain or stiffness as the limiting factor of movement in the ROM with both pain and stiffness present at a high level; and when stiffness was greater than pain, the OMPT felt stiffness as the limiting factor of movement in the end of the ROM with pain at a low level (P > 5/10). Anatomical level (L_1_-L_2_ to L_5_-S_1_) and location (central or unilateral) were chosen, by palpation during PA mobilization, as the most painful and stiffest sites. The slump test was considered as painful (P), when pain was radiating below the knees and not painful (no P) when not.

For PA mobilizations, the OMPT chose spinous process(es) or lamina(e) force application location, the movements components and grades (rhythm and amplitude) varying with his examination findings and the patient’s pain evolution^4,35^, and mobilizations duration, similar to clinical practice treatment. A decision tree used by the OMPT to select the PA mobilizations parameters is available in Fig. 1. PA mobilization grade III corresponds to a large amplitude movement that reaches the end range of motion (ROM), and grade IV to a small amplitude movement at the very end ROM, as defined by Maitland^4^. Total mobilization duration was timed, and primary (more than half the total mobilization time) and secondary (less than half the time) locations of the applied forces on spinous processes (↓) or laminae (left:  and right: ) were gathered (Table 1).

### MRI acquisition

Two lumbar MRI scans were performed for each participant, one before and one after spinal mobilization. All sessions were conducted at the same time of the day (6:00–8:00 PM) to control fluid diurnal variations content in IVDs.

The procedure used for image acquisition was similar to that described by Beattie *et al*.^10^. All images were obtained using a 1.5 Tesla MRI scanner (MAGNETOM Symphony, Siemens AG, Munich, Germany) at the nuclear magnetic resonance department of Grand Hôpital de Charleroi (Site of “Notre-Dame”, Charleroi, Belgium). Multi-element spine coils were used for the T2-weighted and DW images. An abdominal coil was also used for the DW images. Participants entered the scanner head first, with the hips and knees flexed to approximately 30 degrees. Spin echo techniques were used to obtain T2-weighted sagittal and axial views using the parameters described in Table 2. DW image parameters are also summarized in Table 2. For each slice, DW imaging was obtained by applying diffusion gradients in 3 orthogonal directions and the mean $ADC$ was constructed on the basis of averages of signal intensity from 3 directional DW images^10^. The diffusion-weighting $b$-factor was 400 $s\,m{m}^{-2}$, regarded as the best combination of diffusion weighting and signal intensity^10,11,13,36^. There was no inter-slice gap and voxels size of 2.6 × 2.1 × 4.0 $mm$ were used.

**Table 2:** T2- and DW parameters used for MRI. FoV: field of view; TE: echo time; TR: repetition time.

T2-weighted images	
	FoV read: 300 mm
Slices: 13	FoV phase: 100.0%
Dist. Factor: 10%	Slice thickness: 4.0 mm
Orientation: S > T2.1	Base resolution: 384
Phase enc. dir.: H>>F	Phase resolution: 75%
Phase oversampling: 70%	TR: 3500 ms
Flip angle: 150 deg	TE: 93 ms
Fat suppression: none	Averages: 2
Water suppression: none	Concatenations: 1
Antennae: SP3-5	Filter: Distortion corr. (2D)
	Coil elements: SP3-5
**Diffusion-weighted images**	
	FoV read: 400 mm
Slices: 16	FoV phase: 100.0%
Dist. Factor: 10%	Slice thickness: 4.0 mm
Orientation: S > T3.6	Base resolution: 192
Phase enc. dir.: A>>P	Phase resolution: 80%
Phase oversampling: 34%	TR: 3500 ms
Fat suppression: SPAIR	TE: 88 ms
Antennae: SP2-5	Averages: 4
	Filter: Distortion corr. (2D)
	Coil elements: SP3-6

A 3-level modified version^10^ of the grading system initially developed by Pfirrmann *et al*.^37^ was used to identify the presence and extent of IVD degeneration. Intensity (brightness) and T2 signal homogeneity in the central region of midsagittal images was estimated for all IVDs. Hyperintense, homogenous, bright-white NP, with a clear distinction between the AF and NP was graded as 1 (normal); inhomogeneous, gray NP, that can be distinguished from the AF as 2 (intermediate); and inhomogeneous, gray or black NP that cannot be distinguished from the AF as 3 (hypointense). Each participant’s T2-weighted images were evaluated independently by one single investigator (R.F.) and a radiologist with more than 30 years’ experience in the field of musculoskeletal imaging, to classify the IVDs and consensus between the 2 examiners was used to address any classification disagreements^12^.

### Image analysis

**Figure 2:**
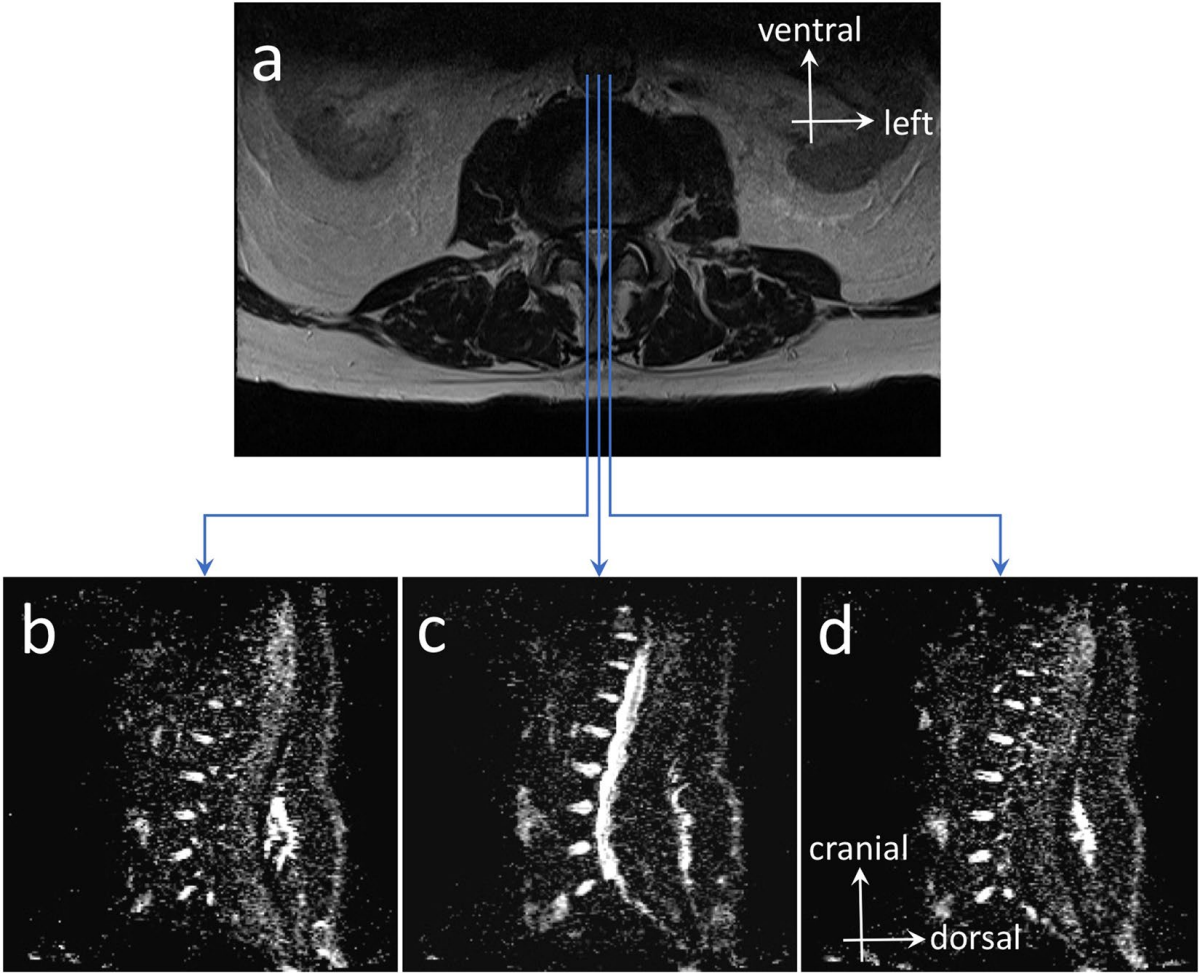
T2-weighted MRI cross section at L_4_-L_5_ IVD and $ADC$ mappings in sagittal medial and parasagittal planes. Position of the 3 section planes are shown on T2 image (**a**) and their resultant $ADC$ mappings in parasagittal right (**b**), sagittal medial (**c**), and parasagittal left (**d**) planes.

**Figure 3:**
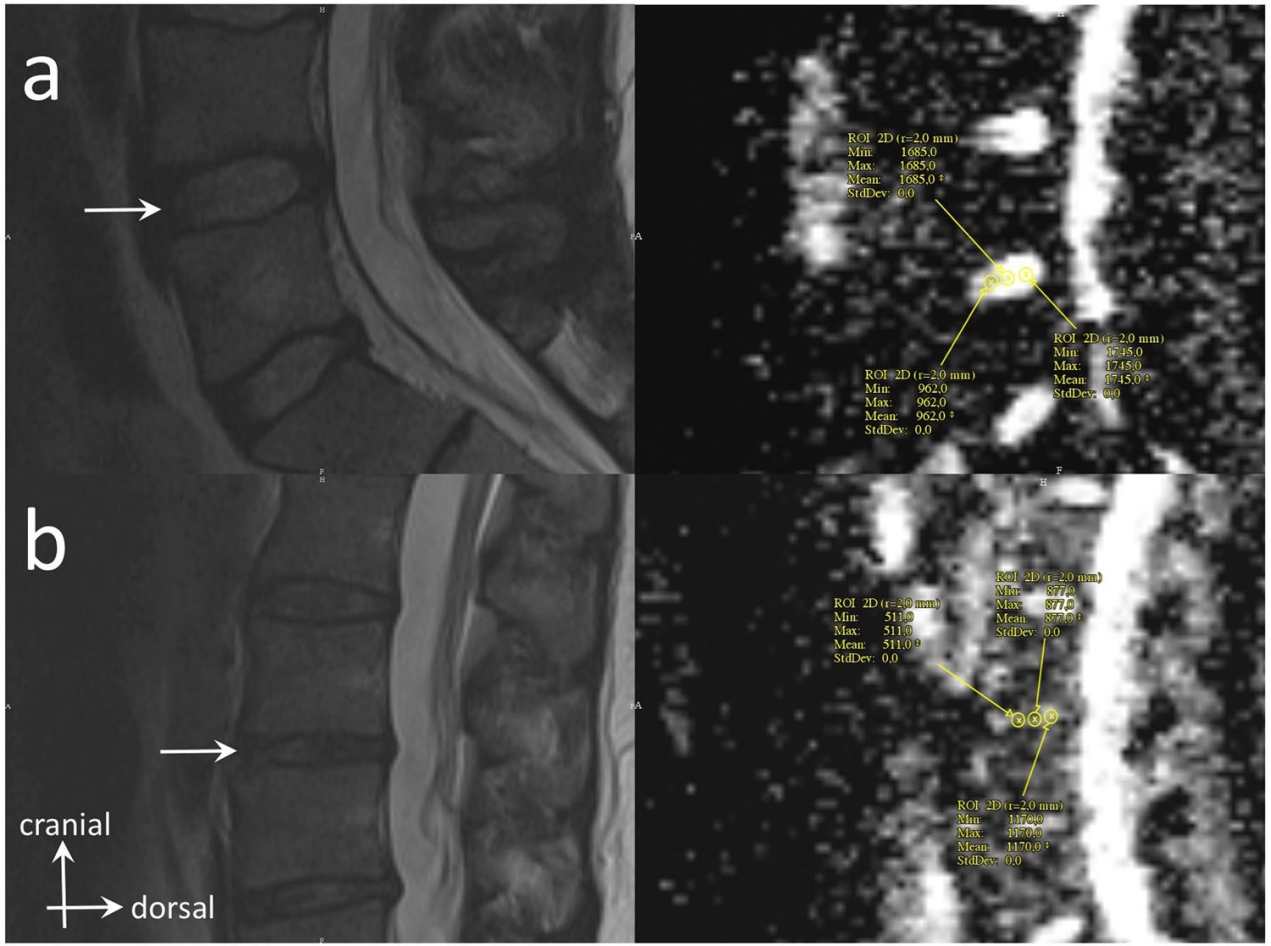
(**a**) T2-weighted image for an IVD classified as Pfirrmann’s grade 1 (arrow, left panel) and the corresponding diffusion-weighted image with the location of anterior, middle, and posterior ROIs used for the computation of $ADC$ values (right panel). (**b**) Similar T2- and diffusion-weighted images for an IVD classified as Pfirrmann’s grade 3.

**Figure 4:**
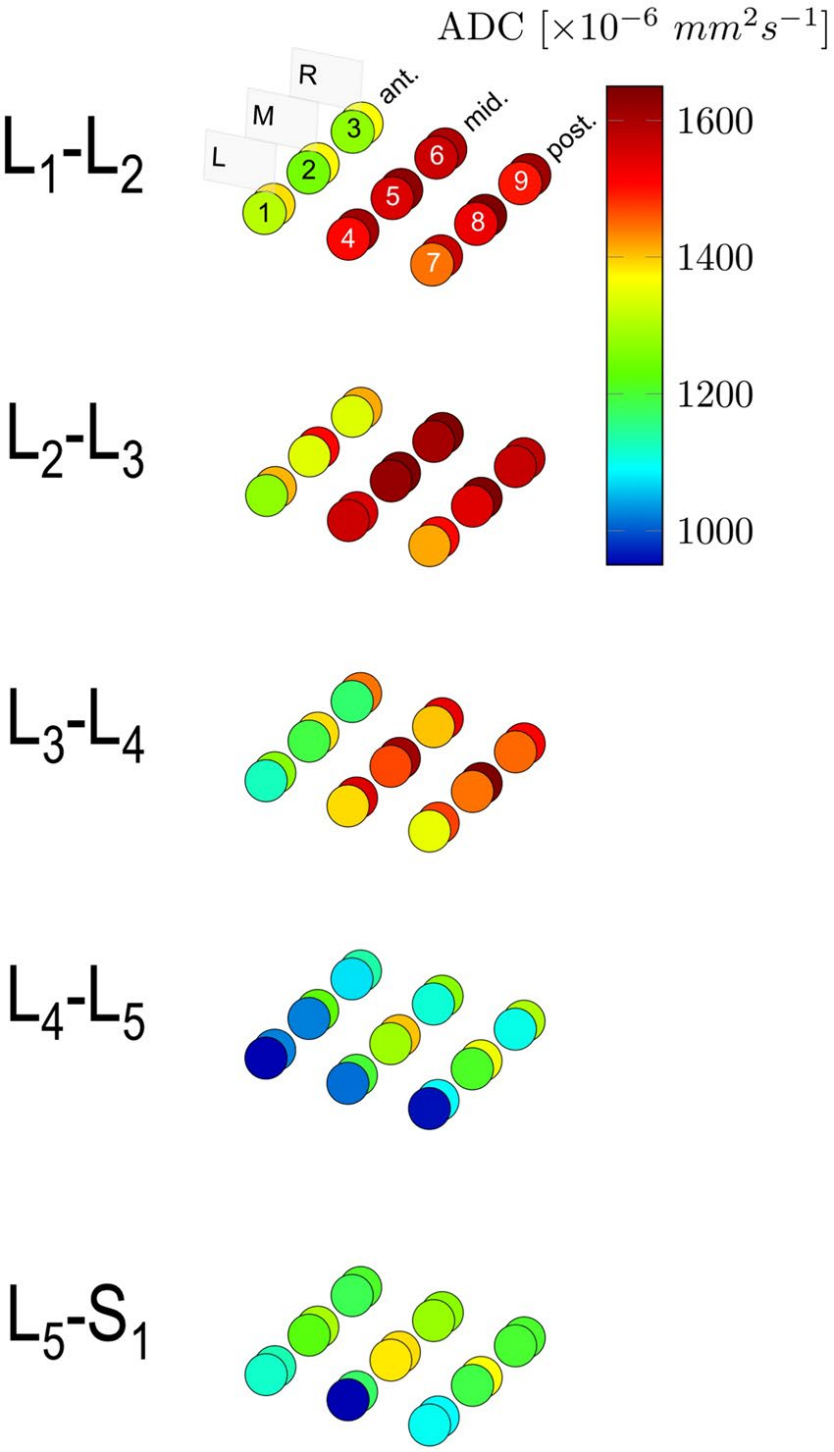
Mean $ADC$ values before and after intervention, for the 9 ROIs (#1 to #9) at the 5 anatomical levels (L_1_-L_2_ to L_5_-S_1_). The color code denotes the importance of $ADC$ values, with cold colors (blue, cyan) for low values and warm colors (red, brown) for high values. Anterior (ant.), middle (mid.) and posterior (post.) portions of the IVDs along the sagittal medial (M, ROIs #2, #5, and #8), parasagittal left (L, ROIs #1, #4, and #7) and right planes (R, ROIs #3, #6, and #9). Values before the intervention are represented by the circles in the foreground and the ones after the intervention in the background.

Diffusion sequences were acquired to quantify the water molecules micro-movements within the lumbar spine $ADC$ IVD and provided water molecules freedom to move images. Maps of the mean $ADC$ were calculated on-line using standard software provided by the MRI manufacturer (Syngo, Siemens Healthcare). After the images were obtained, the files were saved and transferred to a remote workstation for analysis. The radiologist and one investigator (R.F.) interpreted images and calculated $ADC$. The adequate position of the 3 section planes used for $ADC$ measurements were verified by co-registering them to a T2-weighted cross section passing through the IVD (Fig. 2a). $ADC$ measurements were conducted for each IVD in the right parasagittal (Fig. 2b), sagittal medial (Fig. 2c), and left parasagittal planes (Fig. 2d). The adequate position of half-height of each lumbar spine IVD on the $ADC$ map was determined using a T2-weighted cross section passing through the IVD. $ADC$ measurements were computed from 9 specific ROIs of 0.2 $c{m}^{2}$ surface that were selected respectively in the anterior, middle and posterior IVD portions along the sagittal medial (ROIs #2, #5, and #8) and parasagittal left (ROIs #1, #4, and #7) and right planes (ROIs #3, #6, and #9). The ROIs location was determined visually, without using a preset spacing. An example of T2- and diffusion-weighted cross sections of two IVDs classified as Pfirrmann’s grades 1 and 3 are available in Fig. 3a,b, respectively. Mean of anterior ROIs #1 to #3 ($AD{C}_{ant}$), middle ROIs #4 to #6 ($AD{C}_{mid}$), posterior ROIs #7 to #9 ($AD{C}_{post}$) were computed (see Fig. 4). Mean of $AD{C}_{ant}$, $AD{C}_{mid}$, $AD{C}_{post}$ was computed as $AD{C}_{all}$.

### Statistical analyses

All statistical procedures were performed with SigmaPlot software (Version 11.0, Systat Software, San Jose, CA). Data are presented as means and SD and were checked for normality (Shapiro-Wilk) and equal variance tests.

A one-way Repeated Measures Analysis of Variance (RM ANOVA) was conducted to compare the effect of PA mobilizations on pain (VAS and OAS) and trunk mobility results ($TF$, $TE$, $TL{F}_{l}$, and $TL{F}_{r}$). A two-way (level $\times$ treatment) RM ANOVA was conducted to compare the effect of PA mobilizations (treatment) on $ADC$ results in the 5 IVD anatomical levels (level: L_1_-L_2_ to L_5_-S_1_), with a *post hoc* Holm-Sidak method for pairwise multiple comparisons. We did not plan to study the statistical differences in $ADC$ between the anterior, middle, and posterior IVD regions for the different anatomical levels. The effect size (${\eta }^{2}$) was calculated as the sums of squares for the effect of interest (level, treatment and level $\times$ treatment) divided by the total sums of the squares^38^. The benchmarks of Cohen were used to define small (${\eta }^{2}$ = 0.01), medium (${\eta }^{2}$ = 0.06) and large (${\eta }^{2}$ = 0.14) effects^38^. The significance level $\alpha$ was set at 0.05 for all analyses and *post hoc* statistical power was calculated (SigmaPlot, Version 11.0, Systat Software, San Jose, CA) for all pairwise comparisons to allow for interpretation of clinical importance of non-significant results.

Clinical (pain and mobility) and MRI ($AD{C}_{all}$) changes (Δ) between after and before PA mobilizations application were computed as: Δ$VAS$, Δ$TF$, Δ$TE$, Δ$TL{F}_{l}$, Δ$TL{F}_{r}$, and Δ$AD{C}_{all}$. To determine whether Δ$AD{C}_{all}$ correlated with Δ$VAS$, Δ$TF$, Δ$TE$, Δ$TL{F}_{l}$, and Δ$TL{F}_{r}$, a principal component analysis (PCA) was performed with R software (version 3.4.3, FactoMineR and factoextra packages). The Kaiser^39^ rule of eigenvalues greater than 1 and the scree plot^40^ of the percentage of explained variances by each component as a percentage of the total variance were used to determine the number of relevant components.

Box-plots for Δ$AD{C}_{all}$ results according to the modified Pfirrmann’s grades and the anatomical levels were drawn. Bar charts for Δ$AD{C}_{all}$ results according to the anatomical levels, primary level of application and grades of PA mobilizations were drawn.

Test-restest (relative) reliability of $ADC$ measures between 2 MRI scans for one LBP participant (male, 33 years, 183 $cm$, 93 $kg$, pain duration: one week) was estimated using an $ICC$ calculated using R software (version 3.4.3, irr package), based on a single rater/measurement, absolute-agreement, two-way random effects model (ICC(2,1), see Shrout and Fleiss^41^). The investigator (R.F.) was blinded to the slice selections and ROI placement for the test-retest analysis.The participant was sitting on a chair during 15–20 $min$ before and between the 2 measures, and did not receive the lumbar mobilization intervention. Good to excellent relative reliability results were observed for $AD{C}_{all}$, $AD{C}_{ant}$, $AD{C}_{mid}$, and $AD{C}_{post}$, with $ICC$ ranging from 0.86 to 0.98.

Within-participant variability, or absolute reliability, attributable to repeated measures between 2 MRI scans, was assessed by the standard error of measurement percent change ($SE{M}_{ \% }$) calculated as (SEM/Mean) × 100, where SEM is the standard error of measurement and Mean is the mean of all observations from the 2 scans. SEM was calculated as SD × $\sqrt{1-ICC}$, where SD is the standard deviation of the pooled measures of the 2 scans^10^. $SE{M}_{ \% }$ results ranged from 2.1 to 4.7.

## Results

### Classification of T2-weighted signal of nuclear region

Percentage of participants for the 3 grades on the modified Pfirrmann grading system were: 0% for grade 1, 87.5% for grade 2, and 12.5% for grade 3 at L_1_-L_2_; 12.5%, 81.3%, and 6.2% at L_2_-L_3_; 18.8%, 75%, and 6.2% at L_3_-L_4_; 12.5%, 37.5%, and 50% at L_4_-L_5_; 6.2%, 43.8%, and 50%, respectively, at L_5_-S_1_.

### Clinical data

Only PA grades III and IV were chosen by the OMPT, ranging from III− − to IV+ +. The maximal weight change for all participants observed during PA application was 20.9 ± 8 kg (10–30) for grade III, and 22.4 ± 5 kg (19–31) for grade IV. Mean ± SD PA mobilizations total duration was 649 ± 108 $s$. Primary PA mobilizations locations were L_1_ ($n$ = 1), L_3_ ($n$ = 3), L_4_ ($n$ = 7), and L_5_ ($n$ = 5) levels, and secondary locations were only applied on 3 participants at T_11_ ($n$ = 1), L_1_ ($n$ = 1), and L_5_ ($n$ = 1) levels (Table 1). All participants had a DN4 score $<$4, indicating the absence of neuropathic pain. Median (Q1–Q3) QDSA-T was 22 (18.5–26.5), QDSA-S was 13.5 (9.75–16.25), and QDSA-A was 10 (5.75–11.5).

VAS and OAS pain ratings were significantly reduced after mobilization with a large effect size (Table 3). A mean ± SD reduction on VAS of 3.4 ± 1.7 on 10 (62 ± 25%) was observed. Trunk mobility, assessed by $TF$, $TE$, $TL{F}_{l}$, and $TL{F}_{r}$, was significantly increased with medium to large effect sizes (Table 3). A mean reduction of major fingertip-to-floor distance of 6 $cm$ was observed for $TF$, 5 $cm$ for $TE$, 4 $cm$ for $TL{F}_{l}$, and 5 $cm$ for $TL{F}_{r}$.

**Table 3:** One-way RM ANOVA results for pain and trunk mobility and two-way RM ANOVA results for *ADC* (treatment factor).

	Before	After	F	P-value	Power	Effect size (*η*^2^)
	Mean ± SD	Mean ± SD				
Pain (on 10)						
*VAS*	5.4 ± 1.9	2.1 ± 1.5	61.9	<**0**.**001**	1.000	0.510
*OAS*	5.5 ± 1.6	2.3 ± 1.7	61.8	<**0**.**001**	1.000	0.523
Mobility (cm)						
*TF*	28 ± 15	19 ± 13	12.9	**0**.**003**	0.911	0.092
*TE*	62 ± 5	57 ± 6	13.2	**0**.**002**	0.919	0.199
*TLF*_*l*_	50 ± 6	46 ± 6	20.5	<**0**.**001**	0.991	0.157
*TLF*_*r*_	49 ± 8	44 ± 5	14.3	**0**.**002**	0.939	0.130
*ADC*						
*ADC*_*all*_			98.9	<**0**.**001**	1.000	0.026
*ADC*_*ant*_			83.8	<**0**.**001**	1.000	0.041
*ADC*_*mid*_			21.2	<**0**.**001**	0.992	0.014
*ADC*_*post*_			69.4	<**0**.**001**	1.000	0.022

*VAS*: visual analogue scale; *OAS*: oral analogue scale; *TF*: trunk flexion; *TE*: trunk extension; *TLF*_*l*_: lateral flexion left; *TLF*_*r*_: lateral flexion right; *ADC*_*all*_: mean of *ADC*_*ant*_, *ADC*_*mid*_, and *ADC*_*post*_; *ADC*_*ant*_: mean of anterior ROIs; *ADC*_*mid*_: mean of middle ROIs; *ADC*_*post*_: mean of posterior ROIs; significant values are in bold.

### Diffusion of water within IVDs

Mean $ADC$ values before and after intervention for the 9 ROIs at the 5 anatomical levels for anterior, middle, and posterior IVD portions along the sagittal medial, and parasagittal left and right planes are presented in Fig. 4.

A significant mean increase in $AD{C}_{all}$ values was observed after mobilization, with difference of means between 82.1 (change of 5.9%) and 160.7 × 10^−6^ $m{m}^{2}\,{s}^{-1}$ (13.2%) (Tables 3 and 4). Similar significant results were observed in the anterior [$AD{C}_{ant}$ between 99.2 (8.8%) and 205.5 × 10^−6^ $m{m}^{2}\,{s}^{-1}$ (20%)], middle [$AD{C}_{mid}$ between 71.1 (5%) and 151.8 × 10^−6^ $m{m}^{2}\,{s}^{-1}$ (16%)], and posterior portions of the IVD [$AD{C}_{post}$ between 76.1 (6.0%) and 159.8 × 10^−6^ $m{m}^{2}\,{s}^{-1}$ (20.1%)]. Significant differences in $AD{C}_{all}$, $AD{C}_{ant}$, $AD{C}_{mid}$, and $AD{C}_{post}$ were observed at all anatomical levels, except L_5_-S_1_ (Table 4). In addition, no significant difference was observed in $AD{C}_{mid}$ at L_2_-L_3_ (Table 4). The greatest $AD{C}_{all}$ changes were observed at the L_3_-L_4_ and L_4_-L_5_ levels and were mainly explained by changes in $AD{C}_{ant}$ and $AD{C}_{post}$, respectively (Table 4).

**Table 4:** Post hoc results of two-way RM ANOVA for *ADC*, stratified according to the 5 IVD levels.

	Before	After	Difference	% Change (95% CI)	t		P-value
	Mean ± SD (95% CI)	Mean ± SD (95% CI)					
*ADC*_*all*_							
L_1_-L_2_	1437 ± 233 (1188–1685)	1536 ± 231 (1290–1781)	89.9	7.2 (5.3–9.1)	4.3		<**0**.**001**
L_2_-L_3_	1477 ± 196 (1268–1686)	1559 ± 180 (1367–1751)	82.1	5.9 (3.2–8.6)	3.6		<**0**.**001**
L_3_-L_4_	1333 ± 315 (997–1668)	1493 ± 297 (1177–1810)	160.7	13.2 (8.7–17.7)	7.0		<**0**.**001**
L_4_-L_5_	1073 ± 346 (705–1442)	1223 ± 333 (869–1577)	149.9	16.0 (9.6–22.3)	6.6		<**0**.**001**
L_5_-S_1_	1210 ± 356 (830–1589)	1236 ± 338 (876–1597)	26.5	4.1 (−2.4–10.6)	1.2		0.250
*ADC*_*ant*_							
L_1_-L_2_	1277 ± 240 (1022–1533)	1377 ± 248 (1112–1641)	99.2	8.8 (3.3–14.2)	3.3		**0**.**001**
L_2_-L_3_	1320 ± 202 (1105–1535)	1445 ± 190 (1243–1647)	124.9	10.0 (5.9–14.1)	4.2		<**0**.**001**
L_3_-L_4_	1161 ± 298 (844–1478)	1367 ± 265 (1084–1649)	205.5	20.0 (13.3–26.8)	6.8		<**0**.**001**
L_4_-L_5_	991 ± 322 (649–1334)	1130 ± 298 (812–1447)	138.2	16.6 (7.5–25.6)	4.6		<**0**.**001**
L_5_-S_1_	1174 ± 313 (841–1508)	1210 ± 334 (854–1566)	35.7	3.9 (−3.0–10.8)	1.2		0.238
*ADC*_*mid*_							
L_1_-L_2_	1541 ± 252 (1273–1809)	1612 ± 241 (1355–1869)	71.1	5.0 (2.5–7.6)	2.1		**0**.**038**
L_2_-L_3_	1599 ± 173 (1415–1783)	1644 ± 175 (1458–1831)	45.3	3.0 (0.5–5.5)	1.3		0.182
L_3_-L_4_	1420 ± 313 (1087–1755)	1567 ± 274 (1275–1858)	145.7	11.6 (6.1–17.0)	4.3		< **0**.**001**
L_4_-L_5_	1138 ± 358 (757–1519)	1290 ± 358 (909–1671)	151.8	16.0 (5.0–26.9)	4.5		<**0**.**001**
L_5_-S_1_	1293 ± 406 (860-1725)	1277 ± 377 (876–1679)	15.4	1.1 (−7.2–9.4)	0.5		0.674
*ADC*_*post*_							
L_1_-L_2_	1492 ± 273 (1202–1783)	1618 ± 243 (1359–1878)	126.3	9.3 (4.0–14.6)	4.2		<**0**.**001**
L_2_-L_3_	1512 ± 268 (1227–1797)	1588 ± 229 (1344–1832)	76.1	6.0 (1.4–10.6)	2.5		**0**.**013**
L_3_-L_4_	1416 ± 368 (1024–1808)	1547 ± 365 (1158–1936)	131.0	10.1 (5.2–15.1)	4.3		<**0**.**001**
L_4_-L_5_	1090 ± 417 (646–1534)	1250 ± 381 (844–1656)	159.8	20.1 (7.5–32.6)	5.3		<**0**.**001**
L_5_-S_1_	1162 ± 375 (763–1562)	1221 ± 340 (859–1583)	59.1	8.6 (−0.9–18.1)	1.9		0.053

Difference of means (in units of 10^−6^ *mm*^2^ *s*^−1^) and mean change (%) in *ADC* after mobilization. CI: confidence interval; *ADC*_*all*_: mean of *ADC*_*ant*_, *ADC*_*mid*_, and *ADC*_*post*_; *ADC*_*ant*_: mean of anterior ROIs; *ADC*_*mid*_: mean of middle ROIs; *ADC*_*post*_: mean of posterior ROIs; significant values are in bold.

### Relationships between clinical and *ADC* results

**Figure 5:**
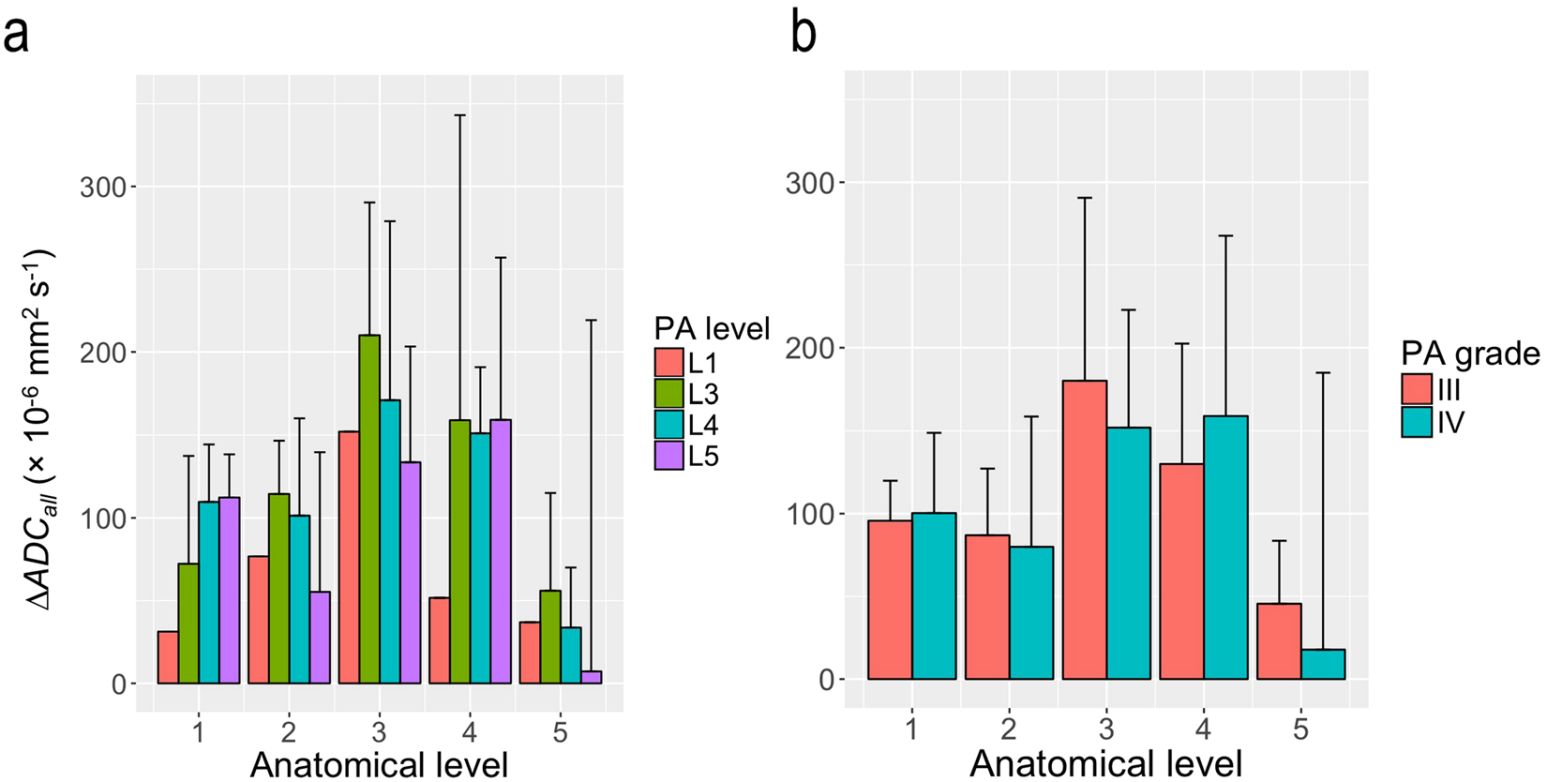
(**a**) Bar chart of mean and SD results for $AD{C}_{all}$ changes after PA mobilizations expressed for each of the 5 anatomical level (1: L_1_-L_2_ to 5: L_5_-S_1_) and the primary level of application of mobilizations (PA level) on the participants (L_1_, L_3_, L_4_ and L_5_). (**b**) Bar chart of mean and SD results for $AD{C}_{all}$ changes after PA mobilizations expressed for each of the 5 anatomical level and the grade of mobilizations (PA grade) applied on the participants (III and IV). These two plots were only drawn for exploratory graphical analyses and the $AD{C}_{all}$ changes observed for PA mobilizations level of application and grades as a function of the anatomical levels were not tested statistically.

Figure 5 presents Δ$AD{C}_{all}$ results for the 5 anatomical levels, according to the primary level of application and PA mobilizations grades. The greatest Δ$AD{C}_{all}$ values observed at the different anatomical levels were not linked to the primary level of PA application (Fig. 5a), and grades III and IV induced similar $AD{C}_{all}$ changes irrespective of the PA mobilization (Fig. 5b).

**Figure 6:**
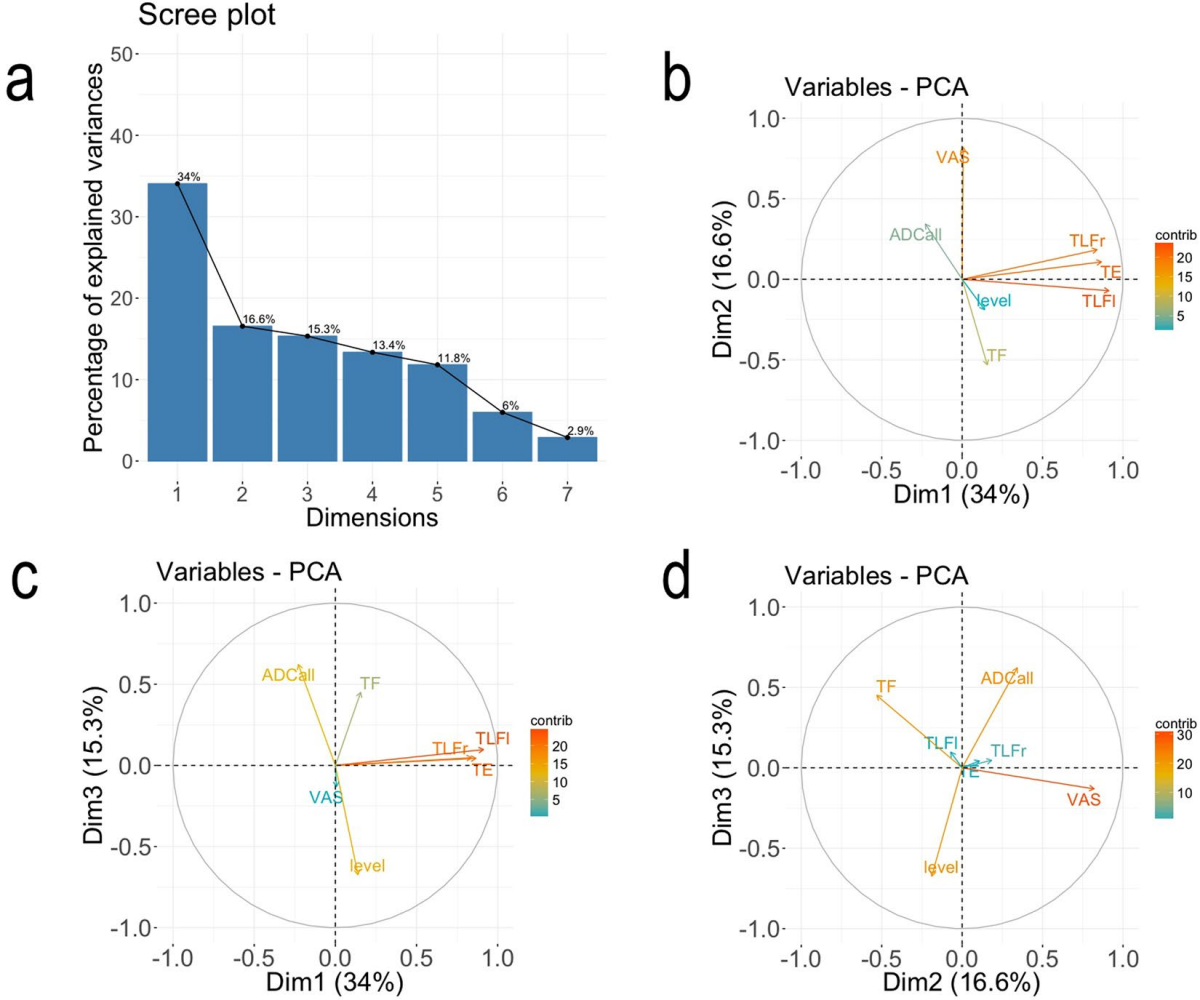
(**a**) Scree plot of percentage of explained variances after PCA. This plot shows the proportion of total variance in the data included in the PCA for each principal component (dimensions), in descending order of magnitude. The scree plot confirms the choice of the first three components to summarize the data (cumulative percentage of variance of 65.9%). (**b**) PCA results: correlation circle for dimensions 1 and 2. (**c**) PCA results: correlation circle for dimensions 1 and 3. (**d**) PCA results: correlation circle for dimensions 2 and 3. The contribution of each variable to the principal axes (‘contrib’) are coded in colors, with cold colors (turquoise blue) showing low contribution and warm colors (orange) high contribution. Dim 1 (mobility), 2 (pain), and 3 (diffusion) denotes the three first dimensions or components, explaining 34, 16.6, and 15.3% of total variance, respectively.

PCA results are presented in Fig. 6 and Table 5. Both the Kaiser^39^ rule [principal component 1 (PC1) = 2.38, principal component 2 (PC2) = 1.16, and principal component 3 (PC3) = 1.07] and the scree plot^40^ (see Fig. 6a and Table 5) indicated that three-factor solution fit the data the best, explaining a cumulative percentage of variance of 65.9%.

**Table 5:** PCA results with eigen analysis and correlation loadings of the 3 first principal components (PCs).

	PC1 (mobility)	PC2 (pain)	PC3 (diffusion)
Eigenvalue	2.38	1.16	1.07
Variance (%)	34	16.6	15.3
Cumulative variance (%)	34	50.6	65.9
Δ*TF*	0.16	**−0**.**53**	**0**.**45**
Δ*TE*	**0**.**87**	0.11	0.04
Δ*TLF*_*l*_	**0**.**91**	−0.07	0.10
Δ*TLF*_*r*_	**0**.**84**	0.18	0.05
Δ*VAS*	0.01	**0**.**82**	−0.13
Δ*ADC*_*all*_	−0.23	**0**.**34**	**0**.**62**
Level	0.14	−0.19	**−0**.**67**

PCA was computed on mobility, pain, and apparent diffusion changes (Δ) between after and before the application of PA mobilizations and also include anatomical level. Values in bold indicate the most important components of each PC.

PCA results are summarized in 3 correlation circles showing vectors pointing away from the origin to represent the original variables (Fig. 6). The angle between the vectors is an approximation of the correlation between the variables. A small angle indicates the variables are positively correlated, an angle of 90 degrees indicates the variables are not correlated, and an angle close to 180 degrees indicates the variables are negatively correlated. In these plots, the contribution of each variable to the principal axes (‘contrib’) are coded in colors (Fig. 6b–d). The main contributing variables to dimension 1 (mobility) were ${\rm{\Delta }}TL{F}_{l}$, ${\rm{\Delta }}TE$, and ${\rm{\Delta }}TL{F}_{r}$. Dimension 2 (pain) was mainly explained by ${\rm{\Delta }}VAS$, ${\rm{\Delta }}TF$, and ${\rm{\Delta }}AD{C}_{all}$ and dimension 3 (diffusion) by anatomical level, ${\rm{\Delta }}AD{C}_{all}$, and ${\rm{\Delta }}TF$. ${\rm{\Delta }}VAS$ was negatively correlated with ${\rm{\Delta }}TF$ (Fig. 6b,d and Table 5) and ${\rm{\Delta }}AD{C}_{all}$ with anatomical level (Fig. 6c,d and Table 5).

## Discussion

The rationale for studying an acute LBP population was based on previous research findings that participants with longer than 2-month symptoms’ duration did not respond as well to manual therapy mobilization^12^. Additionally, while MRI is a technique capable of providing information both on IVD morphology and molecular composition, research efforts should be directed toward characterizing changes directly linked to clinical symptoms^42^.

Our results support previous findings of a simultaneous pain reduction and increase IVD $ADC$ of chronic LBP participants after PA lumbar mobilization^12^ but provide new data concerning the acute phase of disease, and trunk mobility in an older population with higher pain intensity levels. Beattie *et al*.^12^ were the first to explore the short-term effect of oscillating PA mobilizations to the lumbar spinous processes followed by prone press-ups exercises in chronic LBP participants on pain intensity and water diffusion within the IVD NP. They observed two subgroups: “within-session responders” and “not-within-session responders”, based on a reduction of pain of at least 2/10 within-session for the responders. No attempt was made to divide our sample into “within-session responders” and “not-within-session responders” due to our small size sample and that only 4 participants reported less than 2/10 pain reduction.

Mean age of our population was 46 years with a mean pain intensity at baseline of 5.4/10 on VAS. The mean population age studied by Beattie *et al*.^12^ was 26 years with an average pain intensity on a typical day of 3.7/10. The difference in pain intensity between the two studies could not be explained by gender differences, since 9/12 (75%) participants in the “within-session responders” group of Beattie’s study were female and 11/16 (69%) in ours. On the other hand, a difference in body mass index (BMI) could explain it, since higher values are associated with higher pain intensity levels in patients with LBP^43,44^. A mean lower value of 21.0 $kg\,{m}^{-2}$ was observed in “within-session responders” of Beattie’s study compared to 26.6 in ours.

The 62% mean reduction in pain following PA mobilization is higher than that ranging between 33 and 41%, reported in previous investigations when mobilization was applied on the most painful lumbar level, or even at other painful lumbar level and all other lumbar levels^2,45,46^. A potential explanation of this difference could be related to the patients’ groups lower homogeneity of previous investigations that included LBP participants with long pain symptoms duration: up to 3 months^2^, more than 6 months^45^, and even up to 60 months^46^.

Normal IVD is poorly innervated and innervation is restricted to the outer annular layers via branches of sinuvertebral nerve, nerve branches from the ventral rami of spinal nerves, or gray rami communicantes^47^. In contrast, degenerative IVDs display a more important and profound innervation compared to normal IVDs^48^. Furthermore, nociceptive properties of at least some of these nerves are strongly suggested by their immunoreactivity for substance P. These observations are used to defend the hypothesis of the existence of discogenic pain in degenerative IVDs. By definition, discogenic pain is due to a mechanical or chemical irritation of nerves supplying the IVD. Based on our results and those of Beattie and colleagues^10–13^, we believe that the simultaneous reduction in pain observed in patients and increased water diffusion within IVDs is not an epiphenomenon linked to mobilization, and that, on the contrary, these two physiological events are intimately related, directly or indirectly. Although increased $ADC$ does not necessarily equate increased IVD volume, one could hypothesize that increased water diffusion can lead to IVD re-expansion and therefore reduce the mechanical stresses on the large mechanoreceptors nerve fibers. Future studies should evaluate such hypothesis. Increased $ADC$, reflecting the improvement in fluid freedom to flow in the IVD, may contribute to wash away chemical irritants, which may be pain generators in inflamed tissues or degeneration byproducts triggering nerve endings.

On one hand, IVD degeneration starts in the third decade of life, with NP dehydration and changes in its components molecular structures^49^. On the other hand, a link exists between water diffusion in NP, estimated by $ADC$, and visual lumbar IVD degeneration using Pfirrmann’s grading system^50^. Surprisingly, a reduction in $ADC$ values of 4% was observed between normal and moderately degenerated IVDs but severely degenerated IVDs showed 5% larger $ADC$ values than normal IVDs, presumably due to free water in cracks and fissures in the degenerated NP of those IVDs^50^. After a spinal thrust, LBP participants with fewer lumbar degenerated IVDs showed better increased in $ADC$ values than those with many degenerated IVDs^13^. In our study, the majority of IVDs were graded as moderately degenerated at the more cranial anatomical levels and as severely degenerated for more caudal levels, and $ADC$ changes were higher at more cranial levels compared to caudal, with non-significant changes at L_5_-S_1_. Such findings could reflect the differences in outcomes between general lumbopelvic thrust rotational manipulations where greater rotation tends to occur at L_5_-S_1_ with increased $ADC$ values and the application of segmental PA mobilizations, which may not produce similar outcomes in terms of movements and $ADC$ values.

To our knowledge, changes in trunk mobility have never been studied concurrently with changes in pain and water diffusion within the IVDs. Using a PCA, several novel and important observations were made about the relationships between changes in pain, trunk mobility and water diffusion. First, a negative correlation between changes in pain and changes in trunk flexion was observed, but not with changes in extension and lateral flexions. Second, a negative correlation between changes in IVD water diffusion and lumbar anatomic levels was observed. This implies that the greatest $ADC$ changes were observed at more cranial lumbar IVD levels. Previous research reported trunk extension^51–53^ and flexion^52^ mobility to improve or remained unchanged^45,46,51^ after PA mobilization. We showed a significant increase of 29.9 $\pm$ 23% for trunk flexion, 8.1 $\pm$ 8% for trunk extension, 9.9 $\pm$ 8% for left lateral trunk flexion, and 8.9 $\pm$ 8% for right lateral trunk flexion. The significant mean change of 9 $cm$ observed for fingertip-to-floor distance during trunk flexion after PA mobilization in our acute population, was greater than the mean change of 2.7 $cm$ reported by Goodsell *et al*.^46^ in chronic LBP participants. Our results suggest that trunk mobility improvements after PA mobilizations could be larger in acute participants than chronic participants. In comparison to previous studies^11–13^, many differences exist and could explain the findings observed: the strategy of PA mobilization application (duration, force and frequency), the pragmatic patient-centered therapeutic approach used (PA mobilizations applied on the painful anatomical locations with real time pain estimation by an OAS and the selection of grades by the OMPT).

Our $ADC$ values were determined in 80 lumbar IVDs, from L_1_-L_2_ to L_5_-S_1_ levels. An increase in $AD{C}_{all}$ of 7.2% was observed for L_1_-L_2_; 5.9% for L_2_-L_3_; 13.2% for L_3_-L_4_; 16.0% for L_4_-L_5_ and 4.1% for L_5_-S_1_. Beattie *et al*.^12^ observed a mean $ADC$ increase of 4.2% within L_5_-S_1_ IVD in the ‘immediate responder’ group (n=10) after PA mobilization. At all anatomical levels, change in $AD{C}_{all}$ values were greater than $SE{M}_{ \% }$ of 2.1 observed in one participant after 10 minutes of prone lying, which is compatible with the SEM values reported by Beattie *et al*.^11^ on 24 participants after 10 minutes of prone lying and ranging from −3.5 to 3.4%. Therefore, $AD{C}_{all}$ changes observed after PA mobilization must be considered as real changes linked to mobilization and not to measurement errors. It is generally believed that diffusion is the main transport mechanism for small solutes with convection playing a more important role in the transport of larger solutes^17^. DW images provide a characterization of water transport under the combined influence of diffusion and convection. Increased IVD diffusion/convection is thought to be beneficial, while decreased diffusion/convection has been linked to degeneration. Water diffusion within the IVD is influenced by pressure gradients and chemical forces acting on it, as well as structural barriers such as a nuclear “cleft”. Pressure gradients within IVD could be influenced by externally applied forces, such as those generated by manual therapy techniques^13,54,55^. We hypothesize that water diffusion could be related to opening-closure IVD mechanism. This mechanism has been observed *in vivo* by Kulig *et al*.^56^, when applying lumbar spine PA mobilization. A mobilization applied at a given vertebral level results in an extension movement (opening) at this level and on the upper level, and on the contrary a movement of flexion (closure) on the lower level. However, in clinical practice, we suggest following the procedure described by Shah *et al*.^53^ where the most painful segment is targeted first using PA mobilizations in the most painful direction, reproducing the patient pain as described by Maitland^4^ (similar to what we did in the present study).

Correlations were previously described between anatomical levels and $ADC$ values but findings were inconsistent. Some studies showed $ADC$ values to increase^6,36^ or decrease^57^ in more caudal IVDs or to not be correlated with IVD levels^50^. Here, PCA results showed that $AD{C}_{all}$ values tended to decrease in more caudal IVDs. In a more recent study^58^, the influence of age on these relationships was observed, with $ADC$ mean values for young participants (<45 years) increasing from L_1_-L_2_ to L_2_-L_3_/L_3_-L_4_ levels and decreasing to more caudal levels, and decreasing continuously for elderly participants (>45 years). Furthermore, static traction was associated with increased water diffusion within the L_5_-S_1_ IVDs of middle-age individuals, but not in young adults, suggesting age-related differences in the diffusion response^59^.

Today, there is a paucity of research describing the physiologic events associated with analgesia following intervention for LBP^13^. Since $ADC$ is a measure of the magnitude of random (Brownian) diffusion motion of water molecules, it provides information about the IVD physiologic state. Previous studies estimate NP $ADC$ with only one ROI. In the present study, $AD{C}_{all}$ was estimated from the mean of anterior, middle, and posterior IVD portions, which were themselves estimated based on the mean of 3 ROIs (sagittal medial, and left and right parasagittal planes). We believe that our method is more representative of a physiological/physiopathological process of the entire IVD than measures based on a single ROI analyzed in the mid-sagittal scan, since pathologically relevant IVD measurements may be observed in parasagittal or other planes^60^. A significant IVD hyperintense region width was appropriately covered as the IVD volume explored was 15 times greater than that assessed in previous studies^12,13^.

Greatest changes in $AD{C}_{all}$ were observed at L_3_-L_4_ and L_4_-L_5_ levels, and are mainly explained by changes in $AD{C}_{ant}$ and $AD{C}_{post}$, respectively, although this was not tested statistically. More, $AD{C}_{all}$ changes associated to PA mobilizations are not site and grade specific, at least for grades III and IV PA mobilizations at the level of application used in the present study. Note that PA mobilizations were applied between L_3_ and L_5_ in 15 of 16 participants. Since $AD{C}_{ant}$ and $AD{C}_{post}$ were greater than $AD{C}_{mid}$ changes, and taken together with pain decrease, our results suggest that increased peripheral random water molecules motion in the hyperintense IVD center region is implicated in nociceptive response modulation. This observation is important since nerve fibers have been identified in the NP of degenerated IVDs^61^, which may be more likely associated with pain reduction than healthy IVDs that are thought to be innervated only in the annular part. Therefore, it would be interesting to study the influence of these mobilizations, both in NP and annulus fibrosus, according to the 3 orthogonal directions of space (x,y,z) rather than using an average $ADC$ value. Pure water, for the purposes of diffusion is said to be isotropic; this means that the molecules are equally likely to diffuse in any direction. In a biological tissue such as IVD, there may be a preferential diffusion direction, along collagen fibers, and diffusion is said anisotropic. Our methodology did not allow us to study the anisotropic character of water diffusion within IVD. The latter has already been observed previously within lumbar IVDs on healthy young adults^6^, with $AD{C}_{z}$ (diffusion perpendicular to the end-plate) values higher than $AD{C}_{x}$ and $AD{C}_{y}$ (diffusion in the IVD plane). Recently, a promising T2-weighted MRI method based on signal intensity weighted centroid location, i.e. the arithmetic signal intensity mean of all pixels in a ROI, was developed as a biomarker for investigating fluid displacement within the IVD^62^. It would be interesting to apply this method to our images.

This study has some limitations. We stopped the participants’ recruitment before reaching the target sample size estimated a priori to minimize the costs related to MRI and reduce the length of the recruitment period. Our sample was small and composed of participants ranging between 26 and 85 years, which is a quite large range to allow definitive conclusions about $ADC$ changes induced by PA mobilizations. Nevertheless, an heterogeneous age range is representative of a LBP population^1^. Only few IVDs with modified Pfirrmann’s grades 3 were observed in our sample and further studies should confirm if similar results could be expected in participants with higher grades of IVD degeneration. From a methodological point of view, the ROIs selected in more degenerated IVDs could have included anatomical structures located outside the hyperintense region. Also, we could not exclude the fact that $ADC$ differences could be attributed to different slice selections and different participants positions within the scanner between the first and second assessments. In the future, a rigid image registration method could be used with defined slice placement strategies. However, the large IVD volume assessed in our study compensates for this methodological drawback. Regarding the different delay in time spent in sitting position between the first and second MRI assessments and the short distance walked by participants, we assume that it could be a bias in comparison with previous studies^11,13^. In those studies, the observed participants laid during all procedures between the two MRI scans. When lying, the lumbar IVD pressure is much lower compared to sitting and walking^63,64^. In sitting there is significantly less lordosis than prone lying, and significantly more posterior migration of the NP^65^. After 15 minutes of sitting, decreased lumbar IVD height was reported using MRI and stadiometry^66^. Despite these objections and because of our standardized procedure, we believe that $ADC$ changes and positive pain and mobility effects observed after PA mobilizations can be maintained even after sitting and walking a short distance. Another limitation is that we did not plan to study the statistical differences in water diffusion between the anterior, middle, and posterior IVD regions for the different anatomical levels. Future studies could explore water diffusion in different IVD regions with a larger sample. Since the main and most important pathway for diffusion into the NP occurs from capillaries in the vertebral body via diffusion through the cartilaginous endplate^67^, further studies on $ADC$ within IVD should include vertebral endplate morphology evaluation. Finally, no attempt was made to assess participant’s functional disability; the Oswestry Disability Index^68^, considered as the reference standard for measuring degree of disability and estimating quality of life in a LBP participants could have been recorded to complete our sample clinical picture.

In conclusion, the specific application of PA mobilizations at the most painful anatomical locations, and guided in real time by pain perception of acute LBP participants, induced increased water diffusion within all lumbar IVDs, except at L_5_-S_1_ level. This non-specific, multi-level physiological response was associated with pain and mobility improvements.
